# Metronidazole-Induced Pancreatitis: Is There Underrecognition? A Case Report and Systematic Review of the Literature

**DOI:** 10.1155/2019/4840539

**Published:** 2019-06-09

**Authors:** Ibrahim Youssef, Naba Saeed, Mohammad El Abdallah, Kara Huevelhorst, Kais Zakharia

**Affiliations:** ^1^Michigan Medicine, University of Michigan, Ann Arbor, Michigan, USA; ^2^Department of Internal Medicine, Beaumont Health, Dearborn, Michigan, USA; ^3^Department of Radiology, Beaumont Health–Dearborn, Dearborn, Michigan, USA; ^4^Division of Gastroenterology and Hepatology, Department of Internal Medicine, University of Iowa, Iowa City, Iowa, USA

## Abstract

**Introduction:**

Acute pancreatitis (AP) is the most common cause of gastroenterological hospitalization in the USA, with a mortality ranging from 5 to 20%. Up to 80% of cases are caused by cholelithiasis and alcohol abuse. Less common etiologies that need to be explored include hypertriglyceridemia, trauma, ERCP, infections, and drugs. A number of medications are known to cause acute pancreatitis, with 0.3-1.4% of all cases of pancreatitis being drug induced (DIP). Here, we present a case of metronidazole-induced acute pancreatitis.

**Case Summary:**

A 60-year-old female presented with constant severe epigastric pain associated with nausea, vomiting, and anorexia for one day. She had no past medical history of alcohol use or hypertriglyceridemia and was s/p cholecystectomy in the distant past. Symptoms had begun three days after starting metronidazole for* Clostridium difficile* colitis. Lipase was > 396, and CT abdomen revealed peripancreatic fat stranding. She was diagnosed with AP, metronidazole was suspected to be responsible and hence stopped, and supportive management initiated. Her symptoms improved rapidly, and pancreatic enzymes normalized within 2 days. Of note, she had had an episode of acute pancreatitis 3 years ago, also following metronidazole use, with resolution at discontinuation of the drug. She had concurrently been on omeprazole during both episodes.

**Discussion:**

Metronidazole is a commonly used antibiotic and is infrequently reported as a cause of DIP. Our review suggests the possibility of a dose-response and duration-response effect between metronidazole use and occurrence of pancreatitis. The most common presenting symptom and sign was moderate to severe epigastric pain and tenderness, accompanied by nausea/vomiting. Symptoms usually start within 2-7 days of starting the medication and usually resolve 2-5 days after discontinuation of therapy and pancreatitis treatment. The most common causative dose was 1-1.5 g/day. Our review also supports findings by Norgaard et al. suggesting that concurrent use of omeprazole potentiates the risk of metronidazole-induced pancreatitis.

**Conclusion:**

Metronidazole is a commonly used antibiotic that may cause metronidazole-induced pancreatitis, especially if patients are concurrently taking PPIs. Awareness needs to be raised amongst clinicians regarding this association, in order to correctly identify etiology of pancreatitis and discontinue metronidazole promptly when suspected as the causative factor.

## 1. Introduction

Acute pancreatitis (AP) is the most common cause of gastrointestinal hospitalizations in the United States with increasing incidence and costs [1]. Its mortality rate ranges from 5 to 20% depending on the severity of the disease [2]. The most common symptom of pancreatitis is epigastric pain, sometimes radiating to the back, with nausea and vomiting [3]. Atlanta criteria for the diagnosis of AP require two of three of the following: (1) characteristic epigastric abdominal pain, (2) at least three times elevation of serum amylase and/or lipase, and (3) characteristic findings on imaging studies [4]. Gallstones and alcohol abuse are the most common causes of acute pancreatitis accounting for 70-80% of all cases [5]. Other less common etiologies include hypertriglyceridemia, ERCP, hypercalcemia, trauma, infections, and drugs. Identification of the specific etiology, if present, is important as treatment of the underlying etiology can prevent further episodes of AP [6–8].

Only 0.3-1.4% of all cases of AP are attributed to drugs [6–9]. Diagnostic criteria for drug-induced pancreatitis include (1) development of pancreatitis during the treatment, (2) exclusion of other causes, (3) clinical and biochemical improvement with drug withdrawal, and (4) recurrence of symptoms with drug rechallenge [10, 11]. Drugs that induce AP are classified based on the number of reported cases of pancreatitis. Class I includes drugs with >20 reported cases of drug-induced pancreatitis (DIP) with at least one recurrent disease after drug reexposure. Examples include (but not limited to) azathioprine, valproic acid, mesalamine, estrogen preparations, opiates, tetracycline, steroids, trimethoprim/sulfamethoxazole, sulfasalazine, and furosemide. Class II includes drugs with >10 reported cases such as rifampin, octreotide, carbamazepine, acetaminophen, interferon alfa-2b, enalapril, hydrochlorothiazide, cisplatin, and erythromycin. Class III includes drugs with any reported case such as ciprofloxacin, levofloxacin, and azithromycin [12].

Metronidazole is a commonly used antibiotic for treatment of* Clostridium difficile* (*C. difficile*) colitis, trichomoniasis, amebiasis,* Helicobacter pylori* (*H. pylori*), and anaerobic bacterial infections. It is infrequently reported as a cause of DIP. In the above classification, metronidazole was classified under class III. However, we detected 14 cases of metronidazole-induced pancreatitis in the global literature [13–26]. In this study, we present a 15th case of metronidazole-induced pancreatitis.

## 2. Case

A 60-year-old female with a past medical history of hypertension (HTN), diabetes mellitus type 2 (DM2), ulcerative colitis (UC), coronary artery disease (CAD), diastolic congestive heart failure (CHF) with ejection fraction of 60%, acute pancreatitis (1 episode, 2014), and cholecystectomy (in 1990s) presented in 2017 with severe epigastric pain for one day. It was constant, 10/10 in severity, was radiating to the back, and was associated with severe nausea, numerous episodes of nonbloody nonbilious vomiting and anorexia. Symptoms began three days after starting metronidazole for* C. difficile* colitis (developed after treatment with antibiotics for cellulitis). She had no history of alcohol use, hypertriglyceridemia, recent flu-like illness, travel to parasite endemic areas, direct trauma, systemic lupus erythematosus (SLE), vasculitis or other autoimmune diseases. She had no family history of pancreatitis. She had no recent history of endoscopic retrograde cholangiopancreatography (ERCP). She had a 20 pack-year smoking history. Home medications included inhaled albuterol, alprazolam, atorvastatin, clonidine, inhaled fluticasone-salmeterol, losartan, loratadine, montelukast, and omeprazole.

**Figure 1:**
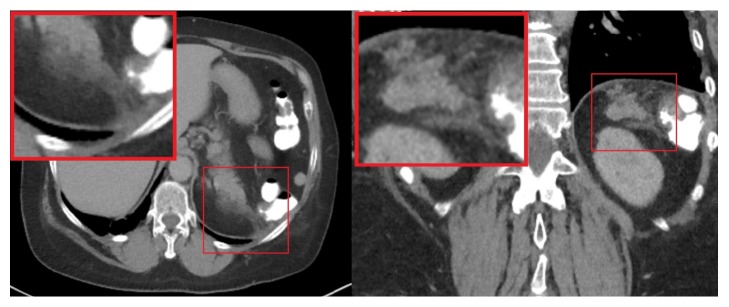
Computed tomography of the abdomen with intravenous and oral contrast. Axial and coronal images demonstrate ill-defined linear increased attenuation adjacent to the tail of the pancreas, representing inflammatory edema in the peripancreatic fat.

Her vital signs at presentation were stable. Her physical exam revealed distended abdomen with severe epigastric tenderness and diminished bowel sounds but no guarding or rebound tenderness. No other abnormal physical exam findings were noted. Labs on admission showed the following: white blood cells (WBC) count was 16,000/*μ*L with neutrophilic predominance and no eosinophilia. Lipase was >396 U/L (amylase not measured). Liver function tests (LFTs) were mildly elevated with total bilirubin 0.7 mg/dL, aspartate aminotransferase (AST) 59 U/L, and alkaline phosphatase (ALP) 135 IU/L. Calcium level was 8.6 mg/dL. Triglycerides level was 69 mg/dL. Antinuclear antibody (ANA) and anti-double stranded DNA (anti-ds DNA) were negative. Ultrasound of the abdomen revealed an 8 mm common bile duct (CBD) s/p cholecystectomy, with no obvious choledocholithiasis. CT of the abdomen and pelvis revealed peripancreatic fat stranding adjacent to the pancreatic tail (Figure 1). A diagnosis of acute pancreatitis was made, with Apache II score of 6 (on admission). The patient was made nil per os (NPO), and fluid resuscitation in addition to pain management was started. Metronidazole was stopped at admission. The symptoms improved rapidly, the lipase downtrended within 2 days (>396→200→134 U/L), and the patient was discharged home.

**Figure 2:**
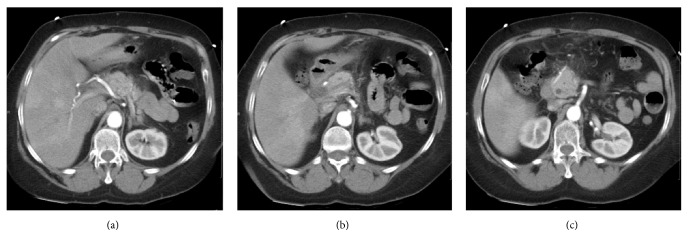
Computed tomography angiography of the chest and abdomen. Contiguous axial images demonstrate mild hazy linear increased attenuation adjacent to the neck (a) and head ((b) and (c)) of the pancreas, representing inflammatory edema.

In 2014, the patient had a similar episode of acute pancreatitis with similar symptoms that developed 4 days after starting metronidazole for* C. difficile* colitis. Lipase at that time was elevated at 808 U/L, and CT angiogram (CTA) of the abdomen and pelvis at that time showed focal edematous pancreatitis of the pancreatic tail (Figure 2). Metronidazole was also stopped at that time, and the patient was made NPO with fluid resuscitation and pain management. Her symptoms improved rapidly, her lipase downtrended within 2 days (808 → 108 U/L), and the patient was discharged home.

## 3. Methods

With the help of an expert research librarian, Medline via PubMed, Ovid, EMBASE, and Cochrane Collaboration databases were searched. Keywords included “metronidazole, Flagyl, acute pancreatitis, and drug-induced pancreatitis”. We also reviewed the references cited in the retrieved articles to obtain all possible cases. 673 abstracts were initially identified, which were then narrowed down to seventeen. Upon full text review, 3 studies were further excluded (Flowsheet 1). The remaining 14 case reports, describing 14 unique patients with 29 events of metronidazole-induced pancreatitis, were included. We then analyzed these cases along with our data, using descriptive statistics. The data was double-checked for accuracy prior to analysis; the results are presented as percentage of the available data. Flow chart 1 (see supplementary material) details how articles were selected for inclusion.

## 4. Results

Including our reported case, our review involves 15 patients who in total had 29 episodes of metronidazole-induced acute pancreatitis. 11 patients were females (73.3%) and 4 patients were males (26.7%). Age ranged between 22 and 74, median 40 years. 7 patients were under 30 years old (46.7%), 5 patients between 30-60 years old (33.3%), and 3 patients were over 60 years old (20%). Of the 11/14 patients from the literature, with prior pancreatitis episodes discussed, 3 patients had 1 episode in total (27.2%), 4 patients had 2 episodes (36.3%), 3 patients had 3 episodes (27.2), and 1 patient had 4 episodes (9%). Indication for the use of metronidazole varied, with vaginitis/vaginal discharge/trichomoniasis being the most common indications in 7 cases (46.6%), followed by* C. difficile* colitis, and diverticulitis in 6 cases (40%), then aspiration pneumonia in 1 case (6.7%) and periodontal abscess in 1 case (6.7%). Metronidazole doses were between 500 mg a day and 2000 mg a day. Overall, 14 patients developed at least one episode of AP within 1 week of starting metronidazole (93.3%), and 1 patient developed AP 11 days after metronidazole use (6.7%). Time since metronidazole use was reported in 27/29 episodes, of which 9 episodes occurred within 1 day of metronidazole use (33.3%), 14 episodes occurred between 2-7 days (51.9%), 3 occurred within the 7-9 days (11.1%), and 1 episode occurred in 38 days (3.7%). All 15 patients had characteristic epigastric pain with or without radiation to the back. Nausea and vomiting were the most common associated symptoms in 11 out of 15 cases (73.3%). Only 2 of the 15 cases had abdominal guarding on exam (13.3%). With respect to lab findings, either lipase or amylase was elevated in all episodes. Amylase was not reported in 8 episodes (27.6%) but was elevated in the remaining 21 cases (72.4%). Lipase was not reported in 12 out of the 29 episodes (41.4%) but was elevated in 14 episodes (48.3%) and was within normal limits in 3 episodes (10.3%). Of all 29 episodes of AP, no imaging was done in 16 episodes (55.2%); positive radiological features of AP were reported in 7 episodes (24.1%), and negative imaging was reported for 6 episodes (20.7%). In 14 out of 15 patients (93.3%), pancreatitis symptoms resolved without complication within a week of stopping metronidazole and starting treatment. In 1 case (6.7%), the patient required a 37-day ICU stay. Pancreatic enzymes normalized in all 15 patients (100%) between 2-10 days after stopping metronidazole and starting treatment.

Interestingly, most patients were on 1000-1500 mg of metronidazole/day and had only been taking it for up to a week before symptom onset. However, the one patient requiring critical care management had been on 2000 mg/day, for 11 days, prior to symptom onset. Details regarding the above cases of metronidazole-induced pancreatitis, found in the literature, are summarized in Table 1.

## 5. Discussion

Our patient met the Atlanta criteria for acute pancreatitis, as she had characteristic symptoms and signs lipase was remarkably elevated, and abdominal CT showed evidence of AP. She also met the criteria for drug-induced pancreatitis, as (1) the episode developed 3 days after starting metronidazole, (2) other etiologies were ruled out, (3) she experienced full resolution after discontinuation of metronidazole, and (4) she had a past history of acute pancreatitis that also occurred shortly after starting metronidazole and resolved after stopping the medication. Given that metronidazole now has 15 reported cases of DIP, and 9 of them were associated with AP recurrence after reintroduction to metronidazole, it can now be considered class II for drug-induced pancreatitis. Although the mechanism whereby it causes DIP is unknown, it is hypothesized that pancreatic injury occurs via free radicals' release during immune-mediated inflammatory responses and via metabolic effects [22].

Our review suggests a few interesting relationships. The one patient who was on higher doses of metronidazole, for greater than 1 week, had the most severe episode of pancreatitis requiring ICU management. This suggests the possibility of a dose-response and duration-response effect.

Next, our patient was taking omeprazole concurrently during her course of metronidazole, both times. In an observational study, Norgaard et al. reported an eightfold increase in pancreatitis from metronidazole when given concurrently with PPIs compared to when given alone [27]. Possible alternate explanations suggested were (i) penetrating PUD requiring PPI use increases risk for pancreatitis and (ii) early symptoms of pancreatitis could be misinterpreted as dyspepsia and treated with a PPI. However, Lancashire et al. also reported a similar effect of metronidazole and PPIs seen in the UK General Practitioner Research Database [28]. While the mechanism remains unknown, this is an important association to recognize as PPI use is rampant in the community. Internal medicine, ob/gyn and gastroenterology providers must all bear this in mind, as metronidazole is also a frequent go-to for the above-mentioned infections.

Lastly, while 73% of patients had nausea and vomiting on presentation, only 13% had abdominal guarding. It is important to remember that during the early phase of pancreatitis, patients may not present with the full spectrum of signs and symptoms and that further investigation with lab work and imaging should be done with any suspicion of pancreatitis, especially if the patient is on metronidazole.

## 6. Conclusion

Metronidazole is a commonly used antibiotic that may cause metronidazole-induced pancreatitis, especially if patients are concurrently taking PPIs. Awareness needs to be raised amongst clinicians regarding this association, in order to correctly identify etiology of pancreatitis and discontinue metronidazole promptly when suspected as the causative factor. Metronidazole now meets class II criteria for DIP, and efforts should be made to identify and report future cases metronidazole-induced pancreatitis. Further studies should be undertaken to identify mechanisms of DIP, and the dose and duration responses to these drugs.

## Figures and Tables

**Table 1:** Metronidazole-induced pancreatitis case reports in the English literature.

First author, year of study	Gender	Age	Number of Episodes	Indication for use of metronidazole (with each episode)	Interval between metronidazole administration and onset of pancreatitis' symptoms (per episode)	Serum amylase, level after the onset of pancreatitis' symptoms (per episode)	Serum lipase level after the onset of pancreatitis' symptoms (per episode)	Imaging findings for acute pancreatitis (per episode)
Plotnick BH, 1985	F	29	1 2	Vaginal discharge	1 day 38 days	1182 U/dL 1727 U/dL	50 U/dL 64 U/dL	N/A N/A

Sanford KA, 1988	F	23	1 2 3 4	Gardnerella vaginalis	9 days 3-7 days 3-7 days 3-7 days	19.07 *µ*kal/L Elevated Elevated Elevated	N/A Elevated Elevated Elevated	Positive N/A N/A N/A

Celifarco A, 1989	F	22	1 2 3	Vaginal infection	24 hours 24 hours 12 hours	250 U/L 280 U/L 1657 U/L	N/A N/A 52.2 U/L	N/A N/A Positive

Corey WA, 1991	F	63	1	Crohn's recurrent rectovaginal fistula	7 days	906 U/L	2148 U/L	Negative

de Jongh FE, 1996	F	45	1 2 3	Vaginitis	N/A N/A 1 day	N/A N/A 120 U/L	N/A N/A N/A	N/A N/A Negative

Sura ME, 2000	F	61	1	Aspiration pneumonia	4 days	566 U/L	245 U/L	Negative

Feola DJ, 2002	F	49	1 2	Trichomoniasis	3-5 days 12 hours	N/A 1397 U/L	N/A 6916 U/L	N/A Negative

Nigwekar SU, 2004	F	46	1 2	Bacterial vaginosis	8 days 8 days	N/A 398 U/L	N/A 2543 U/L	N/A Negative

Tsesmeli NE, 2007	M	31	1	Bloody diarrhea with a history of IBD	3 days	581 U/L	N/A	Positive

Loulergue P, 2008	M	25	1	Pseudomembranous colitis	5 days	317 U/L	665 U/L	N/A

O'Halloran E, 2010	F	25	1 2	Periodontal abscess	3 doses (1 day) 5 doses (2 days)	N/A 810 U/L	N/A N/A	N/A N/A

Cabrera R, 2011	M	74	1	Acute diverticulitis	12 hours	729 U/L	2250 U/L	Negative

Yousaf H, 2012	F	23	1	Trichomoniasis	1 hour	327 IU/mL	876 IU/mL	Positive

Yilmaz M, 2016	M	22	1 2 3	Ulcerative colitis C. *difficile* colitis	3 days 2 days 3 days	N/A 387 IU/L 427 IU/L	N/A 400 IU/L 429 IU/L	N/A N/A Positive

Our case	F	60	1 2	C. *difficile* colitis	4 days 3 days	N/A N/A	808 IU/L 396 IU/L	Positive Positive

N/A: Not applicable.

## Supplementary Materials

Supplementary Materials Flow chart 1. Article selection process.
