# Supplementary material for: Metronidazole-Induced Pancreatitis: Is There Underrecognition? A Case Report and Systematic Review of the Literature
Source: Case Rep Gastrointest Med. 2019 Jun 9;2019:4840539. doi: 10.1155/2019/4840539 (PMC6590551; doi:10.1155/2019/4840539)
Supplement: Supplementary Materials — Flow chart 1. Article selection process. [file 4840539.f1.pptx]

## Slide 1
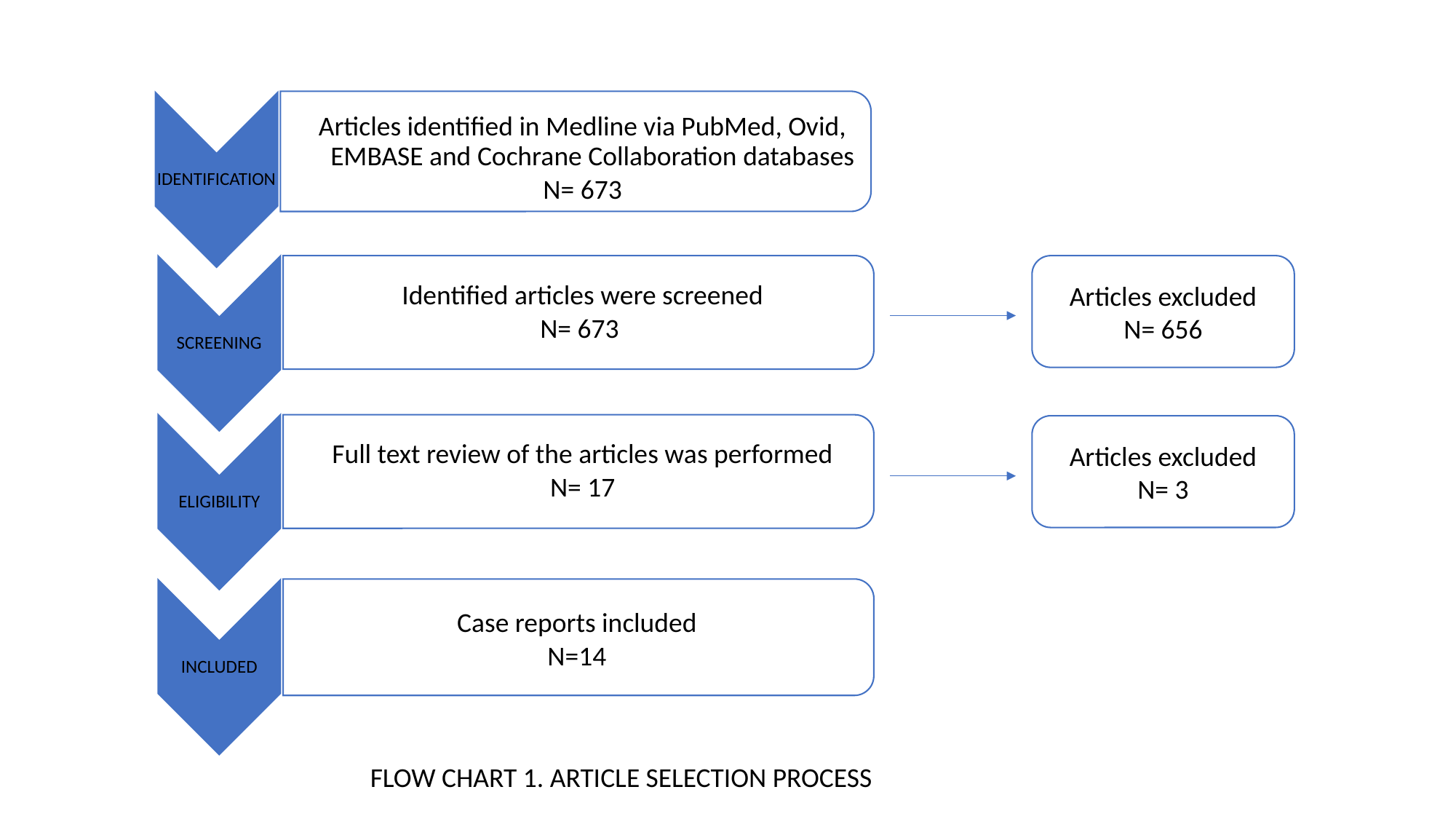

Articles identified in Medline via PubMed, Ovid, EMBASE and Cochrane Collaboration databases
N= 673
IDENTIFICATION
Identified articles were screened
N= 673
Articles excluded
N= 656
SCREENING
ELIGIBILITY
Full text review of the articles was performed
N= 17
Articles excluded
N= 3
Case reports included
N=14
INCLUDED
FLOW CHART 1. ARTICLE SELECTION PROCESS
